# Supplementary material for: Modeling glioblastoma heterogeneity as a dynamic network of cell states
Source: Mol Syst Biol. 2021 Sep 16;17(9):e10105. doi: 10.15252/msb.202010105 (PMC8444284; doi:10.15252/msb.202010105)
Supplement: Supplementary file 5 — Source Data for Figure 3 [file MSB-17-e10105-s001.zip › Figure3A_sourcedata/GSEA_3065/nosigdb_state3.GseaPreranked.1622718426028/RICHARDS_NOWAKOWSKI_SCIENCE_2017_ASTROCYTE_UPREG.html]

Details for gene set RICHARDS\_NOWAKOWSKI\_SCIENCE\_2017\_ASTROCYTE\_UPREG[GSEA]

|  || Dataset | state3 |
| Phenotype | NoPhenotypeAvailable |
| Upregulated in class | na\_pos |
| GeneSet | RICHARDS\_NOWAKOWSKI\_SCIENCE\_2017\_ASTROCYTE\_UPREG |
| Enrichment Score (ES) | 0.46978095 |
| Normalized Enrichment Score (NES) | 2.1988132 |
| Nominal p-value | 0.0 |
| FDR q-value | 1.9642858E-4 |
| FWER p-Value | 0.001 |
Table: GSEA Results Summary

  

Fig 1: Enrichment plot: RICHARDS\_NOWAKOWSKI\_SCIENCE\_2017\_ASTROCYTE\_UPREG      
 Profile of the Running ES Score & Positions of GeneSet Members on the Rank Ordered List

  

| PROBE | GENE SYMBOL | GENE\_TITLE | RANK IN GENE LIST | RANK METRIC SCORE | RUNNING ES | CORE ENRICHMENT || 1 | BCAN |  |  | 0 | 0.883 | 0.0129 | Yes |
| 2 | SCRG1 |  |  | 3 | 0.645 | 0.0220 | Yes |
| 3 | HSP90B1 |  |  | 7 | 0.563 | 0.0299 | Yes |
| 4 | B2M |  |  | 11 | 0.538 | 0.0374 | Yes |
| 5 | PTPRZ1 |  |  | 14 | 0.532 | 0.0449 | Yes |
| 6 | PTN |  |  | 22 | 0.493 | 0.0512 | Yes |
| 7 | CD63 |  |  | 26 | 0.480 | 0.0579 | Yes |
| 8 | CANX |  |  | 27 | 0.479 | 0.0649 | Yes |
| 9 | LAPTM4B |  |  | 31 | 0.473 | 0.0714 | Yes |
| 10 | CD9 |  |  | 32 | 0.473 | 0.0783 | Yes |
| 11 | TIMP1 |  |  | 35 | 0.470 | 0.0849 | Yes |
| 12 | ABHD2 |  |  | 36 | 0.467 | 0.0917 | Yes |
| 13 | GPM6A |  |  | 37 | 0.460 | 0.0984 | Yes |
| 14 | ALCAM |  |  | 45 | 0.440 | 0.1040 | Yes |
| 15 | FGFBP3 |  |  | 51 | 0.431 | 0.1097 | Yes |
| 16 | MALAT1 |  |  | 57 | 0.412 | 0.1151 | Yes |
| 17 | DSEL |  |  | 62 | 0.392 | 0.1203 | Yes |
| 18 | PON2 |  |  | 63 | 0.389 | 0.1260 | Yes |
| 19 | TMBIM6 |  |  | 73 | 0.372 | 0.1303 | Yes |
| 20 | DST |  |  | 80 | 0.365 | 0.1349 | Yes |
| 21 | ITM2B |  |  | 82 | 0.361 | 0.1400 | Yes |
| 22 | MMP16 |  |  | 94 | 0.351 | 0.1438 | Yes |
| 23 | FAT1 |  |  | 95 | 0.350 | 0.1489 | Yes |
| 24 | GPM6B |  |  | 105 | 0.343 | 0.1529 | Yes |
| 25 | MFGE8 |  |  | 107 | 0.341 | 0.1577 | Yes |
| 26 | SCD |  |  | 108 | 0.340 | 0.1627 | Yes |
| 27 | CD81 |  |  | 109 | 0.337 | 0.1676 | Yes |
| 28 | SYT11 |  |  | 111 | 0.335 | 0.1723 | Yes |
| 29 | PDGFRA |  |  | 115 | 0.326 | 0.1767 | Yes |
| 30 | SEMA5A |  |  | 121 | 0.324 | 0.1808 | Yes |
| 31 | BCHE |  |  | 136 | 0.315 | 0.1837 | Yes |
| 32 | SLC16A1 |  |  | 138 | 0.314 | 0.1882 | Yes |
| 33 | TJP1 |  |  | 139 | 0.314 | 0.1928 | Yes |
| 34 | PPIB |  |  | 142 | 0.313 | 0.1971 | Yes |
| 35 | DAD1 |  |  | 144 | 0.313 | 0.2016 | Yes |
| 36 | PCDH9 |  |  | 147 | 0.310 | 0.2058 | Yes |
| 37 | LRP1 |  |  | 152 | 0.309 | 0.2099 | Yes |
| 38 | FADS2 |  |  | 153 | 0.309 | 0.2144 | Yes |
| 39 | IGFBP2 |  |  | 164 | 0.303 | 0.2176 | Yes |
| 40 | PRCP |  |  | 166 | 0.303 | 0.2219 | Yes |
| 41 | ILDR2 |  |  | 171 | 0.299 | 0.2257 | Yes |
| 42 | ZFYVE16 |  |  | 181 | 0.294 | 0.2290 | Yes |
| 43 | TTYH1 |  |  | 183 | 0.293 | 0.2331 | Yes |
| 44 | TMEM106B |  |  | 199 | 0.284 | 0.2354 | Yes |
| 45 | MYO10 |  |  | 206 | 0.280 | 0.2388 | Yes |
| 46 | DAG1 |  |  | 209 | 0.278 | 0.2426 | Yes |
| 47 | LARS |  |  | 214 | 0.276 | 0.2462 | Yes |
| 48 | JAM2 |  |  | 217 | 0.275 | 0.2499 | Yes |
| 49 | SEPT7 |  |  | 221 | 0.274 | 0.2536 | Yes |
| 50 | MLEC |  |  | 223 | 0.273 | 0.2574 | Yes |
| 51 | PCDHGC3 |  |  | 228 | 0.272 | 0.2609 | Yes |
| 52 | TIMP2 |  |  | 230 | 0.271 | 0.2647 | Yes |
| 53 | RCN1 |  |  | 233 | 0.270 | 0.2684 | Yes |
| 54 | LAMB2 |  |  | 237 | 0.269 | 0.2720 | Yes |
| 55 | TNC |  |  | 249 | 0.264 | 0.2745 | Yes |
| 56 | COL6A1 |  |  | 254 | 0.261 | 0.2779 | Yes |
| 57 | SYPL1 |  |  | 261 | 0.258 | 0.2809 | Yes |
| 58 | SSR3 |  |  | 264 | 0.255 | 0.2844 | Yes |
| 59 | HMGCS1 |  |  | 267 | 0.254 | 0.2878 | Yes |
| 60 | PTPRG |  |  | 268 | 0.254 | 0.2915 | Yes |
| 61 | NOVA1 |  |  | 270 | 0.253 | 0.2951 | Yes |
| 62 | NOTCH1 |  |  | 272 | 0.253 | 0.2987 | Yes |
| 63 | ITM2C |  |  | 273 | 0.252 | 0.3023 | Yes |
| 64 | CADM4 |  |  | 299 | 0.244 | 0.3029 | Yes |
| 65 | EDNRB |  |  | 305 | 0.242 | 0.3058 | Yes |
| 66 | LSAMP |  |  | 307 | 0.241 | 0.3092 | Yes |
| 67 | SERPINE2 |  |  | 308 | 0.241 | 0.3127 | Yes |
| 68 | SALL3 |  |  | 312 | 0.240 | 0.3158 | Yes |
| 69 | IL6ST |  |  | 316 | 0.239 | 0.3190 | Yes |
| 70 | ITGAV |  |  | 322 | 0.238 | 0.3218 | Yes |
| 71 | NTRK2 |  |  | 336 | 0.233 | 0.3236 | Yes |
| 72 | MYO6 |  |  | 363 | 0.227 | 0.3238 | Yes |
| 73 | CTNNB1 |  |  | 373 | 0.224 | 0.3260 | Yes |
| 74 | TMED10 |  |  | 378 | 0.223 | 0.3288 | Yes |
| 75 | SEMA6A |  |  | 387 | 0.222 | 0.3310 | Yes |
| 76 | FAM3C |  |  | 406 | 0.217 | 0.3320 | Yes |
| 77 | MSMO1 |  |  | 413 | 0.215 | 0.3344 | Yes |
| 78 | CADM2 |  |  | 416 | 0.215 | 0.3373 | Yes |
| 79 | HSD17B12 |  |  | 432 | 0.212 | 0.3386 | Yes |
| 80 | WSB1 |  |  | 435 | 0.212 | 0.3415 | Yes |
| 81 | LGALS3BP |  |  | 439 | 0.211 | 0.3442 | Yes |
| 82 | PLEKHH2 |  |  | 440 | 0.211 | 0.3473 | Yes |
| 83 | DDR1 |  |  | 454 | 0.208 | 0.3487 | Yes |
| 84 | ITGB8 |  |  | 463 | 0.206 | 0.3508 | Yes |
| 85 | GPC6 |  |  | 468 | 0.204 | 0.3533 | Yes |
| 86 | MAGI1 |  |  | 476 | 0.202 | 0.3554 | Yes |
| 87 | TFPI |  |  | 480 | 0.202 | 0.3580 | Yes |
| 88 | FKBP10 |  |  | 481 | 0.201 | 0.3609 | Yes |
| 89 | UFL1 |  |  | 492 | 0.200 | 0.3626 | Yes |
| 90 | ITGA6 |  |  | 502 | 0.198 | 0.3644 | Yes |
| 91 | LRRC4C |  |  | 509 | 0.197 | 0.3666 | Yes |
| 92 | TTC14 |  |  | 522 | 0.196 | 0.3680 | Yes |
| 93 | DPP6 |  |  | 534 | 0.194 | 0.3695 | Yes |
| 94 | SDC3 |  |  | 539 | 0.194 | 0.3718 | Yes |
| 95 | HIPK2 |  |  | 545 | 0.193 | 0.3740 | Yes |
| 96 | SLC4A4 |  |  | 555 | 0.192 | 0.3757 | Yes |
| 97 | PMP22 |  |  | 556 | 0.192 | 0.3785 | Yes |
| 98 | NLGN3 |  |  | 558 | 0.191 | 0.3812 | Yes |
| 99 | CPE |  |  | 565 | 0.190 | 0.3832 | Yes |
| 100 | PTPRA |  |  | 571 | 0.189 | 0.3854 | Yes |
| 101 | LRRN3 |  |  | 574 | 0.189 | 0.3879 | Yes |
| 102 | SLC2A1 |  |  | 587 | 0.188 | 0.3892 | Yes |
| 103 | SERPINH1 |  |  | 588 | 0.188 | 0.3919 | Yes |
| 104 | EPB41L2 |  |  | 596 | 0.187 | 0.3938 | Yes |
| 105 | OLIG1 |  |  | 602 | 0.186 | 0.3959 | Yes |
| 106 | PRNP |  |  | 609 | 0.185 | 0.3979 | Yes |
| 107 | CLSTN1 |  |  | 614 | 0.184 | 0.4001 | Yes |
| 108 | APC |  |  | 616 | 0.184 | 0.4027 | Yes |
| 109 | SCD5 |  |  | 619 | 0.184 | 0.4051 | Yes |
| 110 | RNF13 |  |  | 635 | 0.181 | 0.4059 | Yes |
| 111 | LIMA1 |  |  | 639 | 0.180 | 0.4082 | Yes |
| 112 | HS2ST1 |  |  | 641 | 0.180 | 0.4107 | Yes |
| 113 | PARP14 |  |  | 664 | 0.176 | 0.4106 | Yes |
| 114 | PLOD2 |  |  | 683 | 0.173 | 0.4110 | Yes |
| 115 | COL11A1 |  |  | 684 | 0.173 | 0.4135 | Yes |
| 116 | NTRK3 |  |  | 687 | 0.173 | 0.4158 | Yes |
| 117 | ERAP1 |  |  | 692 | 0.171 | 0.4178 | Yes |
| 118 | AASS |  |  | 696 | 0.170 | 0.4199 | Yes |
| 119 | FGFR3 |  |  | 708 | 0.169 | 0.4211 | Yes |
| 120 | SLC35F1 |  |  | 711 | 0.168 | 0.4233 | Yes |
| 121 | CSPG5 |  |  | 716 | 0.168 | 0.4252 | Yes |
| 122 | PNISR |  |  | 718 | 0.168 | 0.4276 | Yes |
| 123 | GPRC5B |  |  | 730 | 0.166 | 0.4287 | Yes |
| 124 | SLC1A3 |  |  | 741 | 0.165 | 0.4299 | Yes |
| 125 | PTCH1 |  |  | 743 | 0.165 | 0.4321 | Yes |
| 126 | CST3 |  |  | 749 | 0.164 | 0.4339 | Yes |
| 127 | MEGF10 |  |  | 762 | 0.162 | 0.4349 | Yes |
| 128 | LRIG1 |  |  | 772 | 0.161 | 0.4361 | Yes |
| 129 | CLDND1 |  |  | 773 | 0.160 | 0.4384 | Yes |
| 130 | MCFD2 |  |  | 779 | 0.160 | 0.4402 | Yes |
| 131 | DNASE2 |  |  | 818 | 0.156 | 0.4379 | Yes |
| 132 | PHLPP1 |  |  | 819 | 0.156 | 0.4401 | Yes |
| 133 | PHGDH |  |  | 828 | 0.155 | 0.4414 | Yes |
| 134 | MTRNR2L8 |  |  | 831 | 0.155 | 0.4434 | Yes |
| 135 | TNK2 |  |  | 839 | 0.154 | 0.4448 | Yes |
| 136 | NEAT1 |  |  | 855 | 0.152 | 0.4452 | Yes |
| 137 | HNRNPA2B1 |  |  | 863 | 0.151 | 0.4466 | Yes |
| 138 | SPPL2A |  |  | 870 | 0.151 | 0.4481 | Yes |
| 139 | CYR61 |  |  | 873 | 0.150 | 0.4500 | Yes |
| 140 | GOLIM4 |  |  | 892 | 0.149 | 0.4500 | Yes |
| 141 | SC5D |  |  | 914 | 0.147 | 0.4496 | Yes |
| 142 | RHOBTB3 |  |  | 944 | 0.143 | 0.4482 | Yes |
| 143 | LRP1B |  |  | 953 | 0.142 | 0.4493 | Yes |
| 144 | HTRA1 |  |  | 955 | 0.142 | 0.4513 | Yes |
| 145 | ETV1 |  |  | 963 | 0.141 | 0.4525 | Yes |
| 146 | STAT3 |  |  | 965 | 0.141 | 0.4544 | Yes |
| 147 | CCDC80 |  |  | 966 | 0.141 | 0.4565 | Yes |
| 148 | WSCD1 |  |  | 978 | 0.140 | 0.4572 | Yes |
| 149 | CLU |  |  | 988 | 0.139 | 0.4581 | Yes |
| 150 | JAG1 |  |  | 1011 | 0.138 | 0.4575 | Yes |
| 151 | MLC1 |  |  | 1033 | 0.136 | 0.4569 | Yes |
| 152 | PNPT1 |  |  | 1046 | 0.134 | 0.4574 | Yes |
| 153 | SPECC1 |  |  | 1048 | 0.134 | 0.4593 | Yes |
| 154 | TAPBP |  |  | 1055 | 0.134 | 0.4605 | Yes |
| 155 | PCDH17 |  |  | 1059 | 0.134 | 0.4621 | Yes |
| 156 | ATP9B |  |  | 1063 | 0.134 | 0.4637 | Yes |
| 157 | FJX1 |  |  | 1071 | 0.133 | 0.4648 | Yes |
| 158 | IFI6 |  |  | 1089 | 0.131 | 0.4646 | Yes |
| 159 | ITPR2 |  |  | 1114 | 0.129 | 0.4636 | Yes |
| 160 | PSENEN |  |  | 1123 | 0.128 | 0.4645 | Yes |
| 161 | LDLRAD3 |  |  | 1129 | 0.128 | 0.4657 | Yes |
| 162 | SLC18B1 |  |  | 1159 | 0.125 | 0.4641 | Yes |
| 163 | GJA1 |  |  | 1160 | 0.125 | 0.4659 | Yes |
| 164 | PTPN13 |  |  | 1167 | 0.124 | 0.4670 | Yes |
| 165 | DGKH |  |  | 1182 | 0.123 | 0.4671 | Yes |
| 166 | TMEM132B |  |  | 1194 | 0.122 | 0.4675 | Yes |
| 167 | CSGALNACT1 |  |  | 1202 | 0.121 | 0.4685 | Yes |
| 168 | MAP3K1 |  |  | 1219 | 0.120 | 0.4683 | Yes |
| 169 | IFNAR1 |  |  | 1222 | 0.120 | 0.4698 | Yes |
| 170 | LIFR |  |  | 1260 | 0.117 | 0.4670 | No |
| 171 | COMT |  |  | 1294 | 0.116 | 0.4647 | No |
| 172 | TXNIP |  |  | 1317 | 0.114 | 0.4637 | No |
| 173 | KANK1 |  |  | 1398 | 0.108 | 0.4556 | No |
| 174 | NLGN4X |  |  | 1480 | 0.105 | 0.4474 | No |
| 175 | ZNF621 |  |  | 1486 | 0.104 | 0.4483 | No |
| 176 | HES1 |  |  | 1501 | 0.103 | 0.4481 | No |
| 177 | RNF180 |  |  | 1509 | 0.103 | 0.4488 | No |
| 178 | ACOX1 |  |  | 1544 | 0.101 | 0.4461 | No |
| 179 | FAM181B |  |  | 1629 | 0.096 | 0.4374 | No |
| 180 | ZBTB20 |  |  | 1632 | 0.095 | 0.4385 | No |
| 181 | MEIS1 |  |  | 1663 | 0.093 | 0.4363 | No |
| 182 | MRPS5 |  |  | 1675 | 0.093 | 0.4363 | No |
| 183 | LPIN2 |  |  | 1687 | 0.093 | 0.4363 | No |
| 184 | LRRN1 |  |  | 1690 | 0.092 | 0.4374 | No |
| 185 | C1orf61 |  |  | 1703 | 0.091 | 0.4373 | No |
| 186 | VEGFA |  |  | 1723 | 0.090 | 0.4363 | No |
| 187 | LPP |  |  | 1725 | 0.090 | 0.4375 | No |
| 188 | SMC5 |  |  | 1737 | 0.089 | 0.4375 | No |
| 189 | TSPAN7 |  |  | 1738 | 0.089 | 0.4388 | No |
| 190 | OLIG2 |  |  | 1742 | 0.089 | 0.4397 | No |
| 191 | PTPRD |  |  | 1755 | 0.089 | 0.4396 | No |
| 192 | ARHGEF26 |  |  | 1795 | 0.087 | 0.4361 | No |
| 193 | SPATA6 |  |  | 1796 | 0.087 | 0.4374 | No |
| 194 | SCCPDH |  |  | 1808 | 0.086 | 0.4373 | No |
| 195 | OPHN1 |  |  | 1858 | 0.083 | 0.4326 | No |
| 196 | BMP7 |  |  | 1871 | 0.082 | 0.4324 | No |
| 197 | MAML2 |  |  | 1883 | 0.082 | 0.4323 | No |
| 198 | GABBR1 |  |  | 1892 | 0.081 | 0.4325 | No |
| 199 | ADCY2 |  |  | 1923 | 0.080 | 0.4300 | No |
| 200 | HIBCH |  |  | 1943 | 0.079 | 0.4289 | No |
| 201 | PSAP |  |  | 1988 | 0.076 | 0.4247 | No |
| 202 | NOTCH2 |  |  | 1990 | 0.076 | 0.4257 | No |
| 203 | AXL |  |  | 2020 | 0.075 | 0.4233 | No |
| 204 | CERS6 |  |  | 2062 | 0.073 | 0.4194 | No |
| 205 | TACC1 |  |  | 2084 | 0.073 | 0.4179 | No |
| 206 | SAMD8 |  |  | 2108 | 0.072 | 0.4162 | No |
| 207 | ARAP2 |  |  | 2133 | 0.071 | 0.4143 | No |
| 208 | ST3GAL5 |  |  | 2139 | 0.071 | 0.4147 | No |
| 209 | ABCA3 |  |  | 2201 | 0.068 | 0.4084 | No |
| 210 | TRIM9 |  |  | 2215 | 0.068 | 0.4078 | No |
| 211 | LRRC17 |  |  | 2223 | 0.067 | 0.4079 | No |
| 212 | DPM3 |  |  | 2243 | 0.066 | 0.4066 | No |
| 213 | VPS36 |  |  | 2276 | 0.065 | 0.4037 | No |
| 214 | IDI1 |  |  | 2285 | 0.065 | 0.4036 | No |
| 215 | TRIB2 |  |  | 2296 | 0.065 | 0.4034 | No |
| 216 | ID4 |  |  | 2306 | 0.064 | 0.4032 | No |
| 217 | COG2 |  |  | 2308 | 0.064 | 0.4040 | No |
| 218 | C3orf70 |  |  | 2316 | 0.064 | 0.4041 | No |
| 219 | DNER |  |  | 2341 | 0.063 | 0.4022 | No |
| 220 | RFTN2 |  |  | 2346 | 0.063 | 0.4026 | No |
| 221 | NOTCH3 |  |  | 2367 | 0.062 | 0.4011 | No |
| 222 | IDH1 |  |  | 2376 | 0.062 | 0.4010 | No |
| 223 | EGFR |  |  | 2416 | 0.060 | 0.3972 | No |
| 224 | ADAMTS6 |  |  | 2418 | 0.060 | 0.3979 | No |
| 225 | PDE4B |  |  | 2443 | 0.059 | 0.3959 | No |
| 226 | APCDD1 |  |  | 2511 | 0.057 | 0.3886 | No |
| 227 | SEMA6D |  |  | 2534 | 0.056 | 0.3868 | No |
| 228 | WWC2 |  |  | 2555 | 0.055 | 0.3852 | No |
| 229 | METTL8 |  |  | 2568 | 0.055 | 0.3845 | No |
| 230 | ARHGAP31 |  |  | 2580 | 0.054 | 0.3840 | No |
| 231 | GLDC |  |  | 2660 | 0.051 | 0.3752 | No |
| 232 | MTSS1L |  |  | 2662 | 0.051 | 0.3758 | No |
| 233 | SLC7A11 |  |  | 2682 | 0.051 | 0.3743 | No |
| 234 | HSDL2 |  |  | 2688 | 0.050 | 0.3744 | No |
| 235 | MLLT6 |  |  | 2706 | 0.050 | 0.3731 | No |
| 236 | CNDP2 |  |  | 2721 | 0.049 | 0.3721 | No |
| 237 | NLGN1 |  |  | 2733 | 0.049 | 0.3715 | No |
| 238 | BMPR1A |  |  | 2736 | 0.049 | 0.3720 | No |
| 239 | TNFRSF19 |  |  | 2761 | 0.048 | 0.3698 | No |
| 240 | ZEB1 |  |  | 2764 | 0.048 | 0.3702 | No |
| 241 | ZMYM2 |  |  | 2782 | 0.048 | 0.3689 | No |
| 242 | TSPAN3 |  |  | 2828 | 0.046 | 0.3641 | No |
| 243 | ATP1B2 |  |  | 2838 | 0.046 | 0.3637 | No |
| 244 | SWAP70 |  |  | 2839 | 0.046 | 0.3644 | No |
| 245 | HEPACAM |  |  | 2847 | 0.046 | 0.3642 | No |
| 246 | SLC35A5 |  |  | 2882 | 0.045 | 0.3607 | No |
| 247 | SEL1L3 |  |  | 2895 | 0.044 | 0.3599 | No |
| 248 | ADD3 |  |  | 2929 | 0.043 | 0.3566 | No |
| 249 | ALDH7A1 |  |  | 2940 | 0.043 | 0.3560 | No |
| 250 | CHKA |  |  | 2947 | 0.043 | 0.3559 | No |
| 251 | ABCD3 |  |  | 2974 | 0.042 | 0.3533 | No |
| 252 | NPAS3 |  |  | 2988 | 0.041 | 0.3524 | No |
| 253 | LDHB |  |  | 3031 | 0.040 | 0.3479 | No |
| 254 | ABAT |  |  | 3059 | 0.040 | 0.3452 | No |
| 255 | PAQR8 |  |  | 3112 | 0.038 | 0.3395 | No |
| 256 | DPF3 |  |  | 3123 | 0.038 | 0.3388 | No |
| 257 | FAM19A5 |  |  | 3130 | 0.038 | 0.3387 | No |
| 258 | MED13 |  |  | 3145 | 0.037 | 0.3375 | No |
| 259 | PARD3 |  |  | 3177 | 0.036 | 0.3343 | No |
| 260 | ZDHHC21 |  |  | 3179 | 0.036 | 0.3347 | No |
| 261 | IFI27L1 |  |  | 3203 | 0.036 | 0.3324 | No |
| 262 | CAMK2G |  |  | 3210 | 0.035 | 0.3322 | No |
| 263 | IQSEC1 |  |  | 3221 | 0.035 | 0.3315 | No |
| 264 | HADHB |  |  | 3282 | 0.034 | 0.3248 | No |
| 265 | TLK1 |  |  | 3326 | 0.032 | 0.3201 | No |
| 266 | CASC4 |  |  | 3387 | 0.031 | 0.3133 | No |
| 267 | PDLIM3 |  |  | 3422 | 0.030 | 0.3096 | No |
| 268 | SRPK2 |  |  | 3453 | 0.029 | 0.3064 | No |
| 269 | TAOK3 |  |  | 3466 | 0.029 | 0.3054 | No |
| 270 | SOX8 |  |  | 3470 | 0.029 | 0.3054 | No |
| 271 | IFT88 |  |  | 3481 | 0.029 | 0.3047 | No |
| 272 | PCCB |  |  | 3542 | 0.027 | 0.2978 | No |
| 273 | IGFBP5 |  |  | 3555 | 0.027 | 0.2967 | No |
| 274 | ZNF236 |  |  | 3578 | 0.026 | 0.2945 | No |
| 275 | TMEM170A |  |  | 3589 | 0.026 | 0.2936 | No |
| 276 | PIK3R1 |  |  | 3604 | 0.025 | 0.2923 | No |
| 277 | PBXIP1 |  |  | 3663 | 0.024 | 0.2857 | No |
| 278 | SIRPA |  |  | 3669 | 0.024 | 0.2854 | No |
| 279 | SLC38A3 |  |  | 3682 | 0.023 | 0.2843 | No |
| 280 | MTMR10 |  |  | 3685 | 0.023 | 0.2844 | No |
| 281 | SLC48A1 |  |  | 3691 | 0.023 | 0.2841 | No |
| 282 | ARHGEF6 |  |  | 3694 | 0.023 | 0.2842 | No |
| 283 | NDUFA4 |  |  | 3699 | 0.023 | 0.2841 | No |
| 284 | ARHGAP5 |  |  | 3722 | 0.022 | 0.2817 | No |
| 285 | PARP9 |  |  | 3776 | 0.021 | 0.2756 | No |
| 286 | FERMT2 |  |  | 3823 | 0.020 | 0.2704 | No |
| 287 | APOL2 |  |  | 3845 | 0.019 | 0.2681 | No |
| 288 | NCKAP5 |  |  | 3860 | 0.018 | 0.2667 | No |
| 289 | PTAR1 |  |  | 3879 | 0.018 | 0.2648 | No |
| 290 | FOS |  |  | 3899 | 0.018 | 0.2627 | No |
| 291 | TOB2 |  |  | 3901 | 0.018 | 0.2629 | No |
| 292 | UGP2 |  |  | 3907 | 0.017 | 0.2625 | No |
| 293 | OGFRL1 |  |  | 3920 | 0.017 | 0.2613 | No |
| 294 | ZMAT1 |  |  | 3925 | 0.017 | 0.2611 | No |
| 295 | STK3 |  |  | 3988 | 0.016 | 0.2538 | No |
| 296 | SPRED1 |  |  | 3989 | 0.016 | 0.2540 | No |
| 297 | DCLK2 |  |  | 4033 | 0.014 | 0.2490 | No |
| 298 | HEATR5A |  |  | 4069 | 0.013 | 0.2450 | No |
| 299 | ETV5 |  |  | 4087 | 0.013 | 0.2432 | No |
| 300 | TMEM9B |  |  | 4192 | 0.010 | 0.2307 | No |
| 301 | DTNA |  |  | 4203 | 0.009 | 0.2297 | No |
| 302 | GAS1 |  |  | 4210 | 0.009 | 0.2291 | No |
| 303 | KAT2A |  |  | 4306 | 0.007 | 0.2177 | No |
| 304 | SEPT2 |  |  | 4315 | 0.006 | 0.2168 | No |
| 305 | PDLIM5 |  |  | 4368 | 0.005 | 0.2106 | No |
| 306 | SRGAP2B |  |  | 4375 | 0.005 | 0.2100 | No |
| 307 | BBS2 |  |  | 4407 | 0.004 | 0.2063 | No |
| 308 | SOX9 |  |  | 4503 | 0.001 | 0.1948 | No |
| 309 | SSPN |  |  | 4525 | 0.001 | 0.1923 | No |
| 310 | LAP3 |  |  | 4547 | -0.000 | 0.1898 | No |
| 311 | SASH1 |  |  | 4645 | -0.002 | 0.1781 | No |
| 312 | AGPAT3 |  |  | 4670 | -0.003 | 0.1752 | No |
| 313 | RFX4 |  |  | 4672 | -0.003 | 0.1752 | No |
| 314 | TGFB2 |  |  | 4673 | -0.003 | 0.1752 | No |
| 315 | RNPC3 |  |  | 4683 | -0.003 | 0.1742 | No |
| 316 | PURB |  |  | 4709 | -0.004 | 0.1712 | No |
| 317 | DPP8 |  |  | 4726 | -0.004 | 0.1693 | No |
| 318 | ZCCHC24 |  |  | 4731 | -0.004 | 0.1689 | No |
| 319 | WDR41 |  |  | 4794 | -0.006 | 0.1615 | No |
| 320 | ARHGAP12 |  |  | 4832 | -0.007 | 0.1571 | No |
| 321 | MCC |  |  | 4878 | -0.008 | 0.1518 | No |
| 322 | LRP4 |  |  | 4954 | -0.010 | 0.1429 | No |
| 323 | NRBP2 |  |  | 4967 | -0.010 | 0.1416 | No |
| 324 | PHYHIPL |  |  | 4985 | -0.010 | 0.1397 | No |
| 325 | C2orf72 |  |  | 4986 | -0.010 | 0.1399 | No |
| 326 | NBPF14 |  |  | 5001 | -0.011 | 0.1383 | No |
| 327 | SEPT8 |  |  | 5032 | -0.012 | 0.1349 | No |
| 328 | PLEKHG1 |  |  | 5038 | -0.012 | 0.1344 | No |
| 329 | SFXN5 |  |  | 5066 | -0.013 | 0.1314 | No |
| 330 | PPP2R5A |  |  | 5068 | -0.013 | 0.1314 | No |
| 331 | TEAD1 |  |  | 5118 | -0.014 | 0.1257 | No |
| 332 | USP46 |  |  | 5129 | -0.014 | 0.1247 | No |
| 333 | AHCYL1 |  |  | 5145 | -0.014 | 0.1231 | No |
| 334 | SPARCL1 |  |  | 5152 | -0.014 | 0.1226 | No |
| 335 | NACC2 |  |  | 5231 | -0.017 | 0.1134 | No |
| 336 | OAT |  |  | 5237 | -0.017 | 0.1131 | No |
| 337 | KCNJ16 |  |  | 5242 | -0.017 | 0.1128 | No |
| 338 | GULP1 |  |  | 5255 | -0.017 | 0.1116 | No |
| 339 | TRPS1 |  |  | 5267 | -0.017 | 0.1106 | No |
| 340 | CA2 |  |  | 5274 | -0.018 | 0.1101 | No |
| 341 | OLFM2 |  |  | 5382 | -0.020 | 0.0975 | No |
| 342 | SNAP23 |  |  | 5441 | -0.022 | 0.0908 | No |
| 343 | DOCK1 |  |  | 5496 | -0.024 | 0.0846 | No |
| 344 | CRYL1 |  |  | 5540 | -0.025 | 0.0798 | No |
| 345 | EFCAB2 |  |  | 5572 | -0.026 | 0.0764 | No |
| 346 | IVD |  |  | 5577 | -0.026 | 0.0763 | No |
| 347 | ABI1 |  |  | 5708 | -0.030 | 0.0611 | No |
| 348 | GLUD1 |  |  | 5727 | -0.031 | 0.0594 | No |
| 349 | NTM |  |  | 5738 | -0.031 | 0.0586 | No |
| 350 | ZNF521 |  |  | 5742 | -0.031 | 0.0587 | No |
| 351 | EEPD1 |  |  | 5798 | -0.033 | 0.0525 | No |
| 352 | NEK1 |  |  | 5800 | -0.033 | 0.0529 | No |
| 353 | CTNND2 |  |  | 5812 | -0.033 | 0.0520 | No |
| 354 | RHOJ |  |  | 5872 | -0.035 | 0.0454 | No |
| 355 | EFCAB14 |  |  | 5885 | -0.035 | 0.0445 | No |
| 356 | CDCA7L |  |  | 5899 | -0.036 | 0.0434 | No |
| 357 | TCF7L2 |  |  | 5935 | -0.037 | 0.0397 | No |
| 358 | BDH2 |  |  | 5983 | -0.039 | 0.0346 | No |
| 359 | SESN3 |  |  | 5988 | -0.039 | 0.0347 | No |
| 360 | ZNF639 |  |  | 5998 | -0.040 | 0.0342 | No |
| 361 | DDAH1 |  |  | 6076 | -0.042 | 0.0255 | No |
| 362 | ALDH6A1 |  |  | 6109 | -0.043 | 0.0223 | No |
| 363 | LHX2 |  |  | 6148 | -0.045 | 0.0184 | No |
| 364 | SPRED2 |  |  | 6157 | -0.045 | 0.0181 | No |
| 365 | GATM |  |  | 6161 | -0.045 | 0.0183 | No |
| 366 | SNX5 |  |  | 6177 | -0.046 | 0.0172 | No |
| 367 | RASSF4 |  |  | 6191 | -0.046 | 0.0163 | No |
| 368 | ZC3HAV1 |  |  | 6254 | -0.049 | 0.0095 | No |
| 369 | BAALC |  |  | 6346 | -0.051 | -0.0007 | No |
| 370 | THG1L |  |  | 6378 | -0.052 | -0.0037 | No |
| 371 | RHBDD2 |  |  | 6422 | -0.053 | -0.0081 | No |
| 372 | CLNS1A |  |  | 6427 | -0.054 | -0.0078 | No |
| 373 | MCCC2 |  |  | 6461 | -0.055 | -0.0110 | No |
| 374 | SUCLG2 |  |  | 6466 | -0.055 | -0.0107 | No |
| 375 | NADK2 |  |  | 6473 | -0.055 | -0.0106 | No |
| 376 | ITPK1 |  |  | 6474 | -0.055 | -0.0098 | No |
| 377 | MEST |  |  | 6477 | -0.056 | -0.0092 | No |
| 378 | DUSP6 |  |  | 6502 | -0.056 | -0.0113 | No |
| 379 | QKI |  |  | 6517 | -0.057 | -0.0122 | No |
| 380 | ANXA5 |  |  | 6522 | -0.057 | -0.0118 | No |
| 381 | IRF2 |  |  | 6602 | -0.061 | -0.0205 | No |
| 382 | IMPACT |  |  | 6636 | -0.063 | -0.0235 | No |
| 383 | ROBO3 |  |  | 6639 | -0.063 | -0.0229 | No |
| 384 | FMNL2 |  |  | 6707 | -0.066 | -0.0300 | No |
| 385 | BBX |  |  | 6717 | -0.067 | -0.0301 | No |
| 386 | SNTA1 |  |  | 6734 | -0.068 | -0.0310 | No |
| 387 | PRTFDC1 |  |  | 6760 | -0.069 | -0.0331 | No |
| 388 | ZFP36L2 |  |  | 6774 | -0.070 | -0.0336 | No |
| 389 | RGMA |  |  | 6786 | -0.070 | -0.0339 | No |
| 390 | TGIF1 |  |  | 6888 | -0.075 | -0.0450 | No |
| 391 | ZFP36L1 |  |  | 6893 | -0.076 | -0.0444 | No |
| 392 | CHP1 |  |  | 6896 | -0.076 | -0.0435 | No |
| 393 | SEZ6L |  |  | 6920 | -0.077 | -0.0452 | No |
| 394 | NDRG4 |  |  | 6923 | -0.077 | -0.0443 | No |
| 395 | FYN |  |  | 6924 | -0.077 | -0.0432 | No |
| 396 | SLC25A18 |  |  | 6927 | -0.077 | -0.0423 | No |
| 397 | HEY1 |  |  | 6933 | -0.077 | -0.0418 | No |
| 398 | MAPK1 |  |  | 6941 | -0.078 | -0.0415 | No |
| 399 | GLUL |  |  | 6961 | -0.079 | -0.0426 | No |
| 400 | CAMK2D |  |  | 6988 | -0.080 | -0.0446 | No |
| 401 | CREM |  |  | 7024 | -0.082 | -0.0476 | No |
| 402 | KAT2B |  |  | 7042 | -0.084 | -0.0485 | No |
| 403 | NDUFB5 |  |  | 7052 | -0.084 | -0.0483 | No |
| 404 | ZIC1 |  |  | 7055 | -0.084 | -0.0473 | No |
| 405 | RASSF2 |  |  | 7131 | -0.089 | -0.0551 | No |
| 406 | GNG7 |  |  | 7158 | -0.091 | -0.0569 | No |
| 407 | PEX2 |  |  | 7209 | -0.094 | -0.0616 | No |
| 408 | ENO1 |  |  | 7257 | -0.097 | -0.0658 | No |
| 409 | PSMD10 |  |  | 7263 | -0.097 | -0.0650 | No |
| 410 | MT1X |  |  | 7275 | -0.099 | -0.0649 | No |
| 411 | EFHD2 |  |  | 7282 | -0.099 | -0.0642 | No |
| 412 | UBL3 |  |  | 7332 | -0.103 | -0.0686 | No |
| 413 | ATF7 |  |  | 7364 | -0.105 | -0.0708 | No |
| 414 | PSME2 |  |  | 7389 | -0.107 | -0.0721 | No |
| 415 | GSTM4 |  |  | 7503 | -0.118 | -0.0841 | No |
| 416 | CHPT1 |  |  | 7521 | -0.119 | -0.0844 | No |
| 417 | PHACTR2 |  |  | 7551 | -0.122 | -0.0861 | No |
| 418 | RAB13 |  |  | 7553 | -0.122 | -0.0844 | No |
| 419 | SLC25A5 |  |  | 7582 | -0.126 | -0.0860 | No |
| 420 | FAM120A |  |  | 7624 | -0.130 | -0.0890 | No |
| 421 | PSAT1 |  |  | 7634 | -0.131 | -0.0882 | No |
| 422 | PDXK |  |  | 7701 | -0.139 | -0.0942 | No |
| 423 | JUN |  |  | 7746 | -0.146 | -0.0973 | No |
| 424 | AIF1L |  |  | 7768 | -0.148 | -0.0977 | No |
| 425 | GCSH |  |  | 7778 | -0.149 | -0.0966 | No |
| 426 | RGS20 |  |  | 7818 | -0.154 | -0.0991 | No |
| 427 | NEK6 |  |  | 7896 | -0.165 | -0.1060 | No |
| 428 | SCP2 |  |  | 7919 | -0.170 | -0.1062 | No |
| 429 | ID2 |  |  | 7934 | -0.174 | -0.1053 | No |
| 430 | ID1 |  |  | 7938 | -0.175 | -0.1031 | No |
| 431 | TRIM47 |  |  | 7950 | -0.177 | -0.1019 | No |
| 432 | LITAF |  |  | 8015 | -0.188 | -0.1069 | No |
| 433 | ZMAT3 |  |  | 8086 | -0.201 | -0.1124 | No |
| 434 | SPRY2 |  |  | 8110 | -0.205 | -0.1122 | No |
| 435 | TRAPPC3 |  |  | 8122 | -0.209 | -0.1105 | No |
| 436 | ENHO |  |  | 8124 | -0.210 | -0.1075 | No |
| 437 | MPC1 |  |  | 8135 | -0.213 | -0.1056 | No |
| 438 | FHL1 |  |  | 8139 | -0.214 | -0.1029 | No |
| 439 | HOPX |  |  | 8211 | -0.231 | -0.1081 | No |
| 440 | CNN3 |  |  | 8228 | -0.234 | -0.1066 | No |
| 441 | MRFAP1 |  |  | 8239 | -0.237 | -0.1043 | No |
| 442 | CXXC5 |  |  | 8266 | -0.245 | -0.1039 | No |
| 443 | VEGFB |  |  | 8283 | -0.254 | -0.1021 | No |
| 444 | STK17A |  |  | 8300 | -0.258 | -0.1003 | No |
| 445 | RHOA |  |  | 8302 | -0.259 | -0.0967 | No |
| 446 | ADI1 |  |  | 8320 | -0.263 | -0.0949 | No |
| 447 | GNG12 |  |  | 8323 | -0.264 | -0.0913 | No |
| 448 | MT3 |  |  | 8329 | -0.265 | -0.0880 | No |
| 449 | PEA15 |  |  | 8347 | -0.272 | -0.0861 | No |
| 450 | NDUFB3 |  |  | 8362 | -0.278 | -0.0837 | No |
| 451 | SRI |  |  | 8395 | -0.292 | -0.0833 | No |
| 452 | PKM |  |  | 8420 | -0.301 | -0.0818 | No |
| 453 | LYPD1 |  |  | 8460 | -0.321 | -0.0819 | No |
| 454 | SAT1 |  |  | 8533 | -0.354 | -0.0854 | No |
| 455 | FABP7 |  |  | 8536 | -0.354 | -0.0805 | No |
| 456 | S100B |  |  | 8562 | -0.373 | -0.0781 | No |
| 457 | DBI |  |  | 8571 | -0.383 | -0.0734 | No |
| 458 | GNG5 |  |  | 8575 | -0.384 | -0.0682 | No |
| 459 | GFAP |  |  | 8610 | -0.413 | -0.0663 | No |
| 460 | MT2A |  |  | 8650 | -0.456 | -0.0644 | No |
| 461 | S100A13 |  |  | 8652 | -0.457 | -0.0578 | No |
| 462 | POLR2L |  |  | 8664 | -0.468 | -0.0523 | No |
| 463 | TOMM7 |  |  | 8666 | -0.470 | -0.0456 | No |
| 464 | SNX3 |  |  | 8668 | -0.473 | -0.0388 | No |
| 465 | RHOC |  |  | 8675 | -0.484 | -0.0325 | No |
| 466 | SPATS2L |  |  | 8691 | -0.509 | -0.0269 | No |
| 467 | RAB31 |  |  | 8696 | -0.514 | -0.0198 | No |
| 468 | FABP5 |  |  | 8699 | -0.518 | -0.0125 | No |
| 469 | S100A16 |  |  | 8714 | -0.555 | -0.0061 | No |
| 470 | LGALS1 |  |  | 8744 | -0.718 | 0.0008 | No |
Table: GSEA details [plain text format]

  

Fig 2: RICHARDS\_NOWAKOWSKI\_SCIENCE\_2017\_ASTROCYTE\_UPREG: Random ES distribution      
 Gene set null distribution of ES for **RICHARDS\_NOWAKOWSKI\_SCIENCE\_2017\_ASTROCYTE\_UPREG**
